# Former Food and Agro-Industrial By-Products in Dairy Cow Diets: Effects on Milk Quality and Cheese Production

**DOI:** 10.3390/ani15081113

**Published:** 2025-04-11

**Authors:** Ludovica Maria Eugenia Mammi, Francesca Ghiaccio, Elisa Benini, Carla Giuditta Vecchiato, Isa Fusaro, Giovanni Buonaiuto, Damiano Cavallini, Alberto Palmonari, Giorgia Canestrari, Riccardo Colleluori, Andrea Formigoni

**Affiliations:** 1 Department of Veterinary Medical Sciences, University of Bologna, 40064 Ozzano Emilia, Italy; ludovica.mammi@unibo.it (L.M.E.M.); carla.vecchiato2@unibo.it (C.G.V.); giovanni.buonaiuto@unibo.it (G.B.); damiano.cavallini@unibo.it (D.C.); alberto.palmonari2@unibo.it (A.P.); giorgia.canestrari@unibo.it (G.C.); riccardo.colleluori2@unibo.it (R.C.); andrea.formigoni@unibo.it (A.F.); 2 Faculty of Veterinary Medicine, University of Teramo, 64100 Teramo, Italy; ifusaro@unite.it

**Keywords:** former food, wheat distiller grain, circular economy, cheese quality, dairy cow, livestock environmental sustainability

## Abstract

The use of former foodstuff and by-products in cow diets could improve the environmental sustainability of livestock. However, knowledge about the effects of these feeds in ruminant diets on the quality of dairy products is lacking. This study investigated the effects of integrating wheat distillers’ grain with solubles (WDGS) and a former foodstuff product (FFP) into the diet of dairy cows on milk and cheese quality. The environmental impact of the two feeding systems has been evaluated by LCA. A double crossover design was set up with 84 Holstein cows, alternating a control diet and a circular one. In the circular diet, traditional feeds were partially replaced with 4 kg/d WDGS and 3 kg/d FFP. The results showed no effects on dry matter intake and milk yield, while fiber digestibility improved. A reduction in the milk fat content and a modification of the fatty acid profile of milk and cheese were observed. Conversely, cheese yield, composition, and sensory attributes were not affected. The environmental impact of the diet was significantly reduced in terms of land occupation, net fresh water, and global warming potential. These findings suggest that these feeds can be safely included in dairy cow diets. This approach could significantly contribute to reducing greenhouse gas emissions, enhancing the sustainability of the dairy system.

## Simple Summary

Currently, about one-third of the global food production is lost or wasted along the supply chain, leading to the inefficient use of valuable resources such as land, water, and energy. Redirecting these by-products into animal feed can significantly decrease the reliance of livestock systems on conventional feedstuffs, reducing food waste and mitigating the competition for land use. This study confirms the possibility of using former food and agro-industrial products in the diet of dairy cows and highlights the strong positive impact of this approach on the environmental sustainability of livestock.

## 1. Introduction

The growing availability of by-products coming from several food processing industries has long attracted the interest of animal nutrition because of the numerous environmental, economic, and nutritional advantages resulting from their use [1,2]. In addition to the common industrial by-products (e.g., wheat distillers grain with solubles, beet pulps, soyhulls, wheat bran, etc.), bakeries, grain industries, and supermarkets also generate large amounts of leftovers [1], which mainly consist of products remained unsold (e.g., pasta, sliced bread, cookies, croissants, cakes, and pastries) due to manufacturing and packaging defects [3,4]. According to FAO [5], these products are identified as food loss (FL), defined as “the outcomes of managerial and technical limitations of the early stage of production”. Conversely, food waste (FW) has been defined as losses that occur at the end of the food chain, such as catering and household waste. This specification is fundamental because it determines the possible further utilization of these different compounds. Indeed, according to European Regulation [6], FW cannot be used for feed production due to safety reasons, while FL is listed in the EU Catalogue of Feed Materials as a “former foodstuff” (FFP) and can be used as an ingredient in animal feed [6,7].

According to FAO [5], approximately one-third of the food for human consumption produced globally is lost or wasted along the supply chain. Moreover, food waste and by-products were assessed as contributing to over 20% of the total global production of greenhouse gases, including methane (CH_4_), nitrous oxide (N_2_O), and carbon dioxide (CO_2_), with emissions amounting to 3.3 billion tons of CO_2_ per year, all of which significantly impact climate change [8]. Recent studies have emphasized that the reduction in food waste through its use in animal feed can significantly contribute to lowering greenhouse gas emissions, especially in intensive livestock systems, which are among the major contributors to environmental impact [9].

Minimizing these losses is therefore an important way of improving global food security and the management of land, water, and energy resources in the food production system. The circular economy approach has been addressed as one of the possible instruments to improve and optimize feed and food production systems [10]. This approach aims to retain the value of products, materials, and resources in the economy for as long as possible, thereby reducing losses and waste. Specifically, the adoption of circular strategies in the agri-food sector is increasingly promoted to enhance sustainability while ensuring the efficient use of resources and preventing waste accumulation [11].

Food waste, in this context, plays a crucial role and must be managed effectively across various stages of the value chain to promote sustainability [12]. In particular, the valorization of former food (FFP) and agro-industrial by-products represents an attractive opportunity for the sustainable and competitive development of the food and feed industrial sectors [13,14]. Moreover, incorporating these compounds into animal nutrition can significantly reduce reliance on traditional feedstuffs like cereals and soybeans, which are typically cultivated on arable land. Utilizing alternative feed sources, such as food waste and by-products, has been recognized as a key strategy to improve the sustainability of livestock farming while reducing the competition for land between food and feed production [9]. This shift not only lessens the competition between food and feed but also potentially decreases the pressure on land use, enabling the reallocation of these lands for more sustainable purposes, such as reforestation and the cultivation of crops for human consumption [15]. The use of former food products in animal feeding is already well descripted, especially in monogastric [15,16,17]. However, in ruminant nutrition, especially in dairy cows, there is a lack of information regarding their effects on production quality. Feeding FFP and agro-industrial by-products to dairy cows might be a useful option to replace the large quantities of human-edible foodstuffs (such as cereals and soybeans), which are currently used for cow feeding [18,19]. Moreover, many of these “circular” feedstuffs are characterized by high crude protein (CP) content and low ruminal degradability; such characteristics makes them highly palatable and quite desirable in the feeding of dairy cattle [20].

Previous research has examined the effect of supplementing various agro-industrial by-products and former foodstuff on milk production and feed intake or rumen health [18,21,22,23], but to our knowledge, no research has investigated the effects of these products on cheese production and quality. One of the main issues that limits the inclusion of FFP and WDGS in dairy cow diets is represented by the high variability of their nutrient and chemical composition, which strictly depends on the production process and site of origin [1,2,23]. A deeper knowledge of these aspects is therefore fundamental to avoid any negative impact on the quality and final characteristic of cheese.

Having data on the effect of such products on milk and cheese production may be very relevant, particularly for countries like Italy, where 46.8% of the milk produced is transformed into cheese [24], and where maintaining high cheese quality is fundamental.

For these reasons, this study aimed to evaluate the impact of the inclusion of solid former foodstuff feed (FFP) and a wheat wet distillers grain with solubles (WDGS) in the diet of dairy cows, focusing on their effects on milk composition and cheese quality, while also considering their role in improving feed sustainability. For this purpose, two groups of lactating Holstein cows have been fed a diet including 4 kg/d WDGS + 3 kg/d FFP, and the effects of this diet have been evaluated on milk and final cheese quality.

## 2. Materials and Methods

### 2.1. Experimental Design, Animals, and Feeding

The research was performed at the experimental dairy farm of the University of Bologna, situated in Ozzano dell’Emilia (Bologna) in the northern part of Italy. The entire herd was involved in a double crossover trial, where traditional starch and protein sources were partially replaced with sustainable “circular” feeds coming from the grain industry.

At the beginning of the trial, the herd, composed of 84 lactating Italian Holsteins cows averaging 709.34 ± 88.99 kg of body weight, 192.33 ± 107.9 days in milk (DIM), 1.96 ± 1.06 number of lactations, and 34.16 ± 10.79 kg/d of milk production, was divided into two homogeneous groups. The animals were housed in two comparable pens with straw-bedded cubicles and milked twice a day (at 6 A.M. and 5 P.M.) in a double-5 herringbone milking parlor equipped with a system for recording individual milk production, composition, and body weight (Afimilk Information Management System; Kibbutz Afikim, Israel). Each cow was equipped with a neck collar (Heat Time Pro, SCR; Netanya, Israel; [25]) for continuous recording of activity and to measure rumination time (min./d).

A cooling system, composed of fans and sprinklers, was automatically activated based on the temperature and humidity index (THI) that was recorded continuously by environmental probes located in the barn (CMP Impianti S.r.l., Viadana Bresciana, Italy). During the trial, the average THI was 72.64 ± 5.28.

Two dietary treatments, composed of “circular” (WDGS + FFP) or “traditional” (CTR) feeds, were alternatively offered to each group in 4 experimental periods (2 CTR and 2 WDGS + FFP). (Table 1). Each experimental period was composed of 4 weeks of adaptation and 1 week of data recording and cheese production, for a total of 20 weeks of trial.

Diets’ ingredients and composition are described in Table 2. The control diet included traditional starch and protein sources, while the experimental one (WDGS + FFP) included a combination of wet wheat distiller grain (WDGS) and a pelleted former foodstuff feed (FFP) (4 kg/d WDGS + 3 kg/d FFP), both provided by a feed company specialized in the processing of bakery industry leftovers (Dalma spa; Marene, Italy). The FFP was composed of bakery industry waste, such as pasta, bread, biscuits, and snacks, no longer intended for human consumption of this material. All ingredients are present in EU Catalogue of Feed Materials, as defined by EU regulation 2017/1017 [6]. The products composing the FFP were mainly sourced through large-scale retail trade and pre-selling production stages, as defined by EU regulation 2022/1104 [26]. The FFP is produced and distributed by Dalma spa, which manages all stages of its production. The FFP contains no other non-food ingredients, and the specific recipes, unpacking methods, processing methodologies, and mixing information are protected under the patent rights of the producer. FFP included in the diet was characterized by 41.33 ± 2.03% DM starch content, 11.88 ± 0.58% DM protein, and 6.00 ± 0.5% DM for fat content, as detailed in Table 3.

The inclusion rates for WDGS + FFP were based on Mammi et al. [23], which demonstrated that supplementing with 4 kg/d of WDGS and 3 kg/d of FFP could replace 4 kg/head/day of dry matter from traditional cereals and protein sources without adversely affecting health or production.

Diets, including all dry and nonfermented components, were formulated using the Agricultural Modeling and Training Systems software (AMTS, v. 14.4.0) simulating those used in the Parmigiano Reggiano cheese production area of Italy [27]. Rations were offered as partial mixed rations (PMR). PMR was prepared for each treatment once a day (between 7 and 8 A.M.) and distributed once a day to both groups immediately after preparation using a mixer wagon (Zago Mixer; Padova, Italy). Animals had continuous ad libitum access to potable water across 6 watering troughs for each group. In Table 4, it is reported that the fatty acid profile of the experimental feeds (CTR, FFP and WDGS) and the total amount of fatty acids in the diets (CTR and WDGS + FPP) derived from the amount of inclusion of the experimental preparations tested (CTR, WDGS and FPP).

The feedstuffs included in the diets were sampled at the beginning of each period, while PMR samples and hay were collected once a week for the entire trial. Additionally, feed residuals were sampled 2 times for each experimental week before the distribution of the fresh PMR.

### 2.2. Feed Analysis

All feed samples were analyzed in the laboratories of the Animal Production and Food Safety (SPASA; DIMEVET, University of Bologna), which are accredited according to UNI EN ISO 9001:2015 [28]. The samples were first dried in a forced-air oven at 65 °C for 48 h for DM determination and subsequently grinded with Cyclotec™ 1093 Sample Mill, (FOSS Tecator, Hoganas, Sweden) until they reached 1 mm particle size.

The hay and PMR samples were analyzed by NIRs and the WinISI II Project Manager v1.50 software (FOSS, NIRSystem 6500, DK-3400; Hillerød, Denmark) with equipment for moisture, crude protein, starch, ash-corrected α-amylase–treated neutral detergent fiber with addition of sodium sulfite (aNDFom), acid detergent fiber (ADF), acid detergent lignin (ADL), undigested NDF (uNDF), and ash.

The feedstuff samples (CTR, FFP, and WDGS) were analyzed by wet chemical analysis as described by Mammi et al. [23]. The determination of nutrient was performed according to the following AOAC procedures [29]. The crude protein (CP) content was analyzed following the AOAC method 984.13A, using a Kjeldahl nitrogen analyzer (Gerhadt Vapodest 50, Gerhardt GmbH; Königswinter, Germany), the nitrogen fraction estimated as soluble protein, NDIP (neutral detergent insoluble protein), and ADIP (acid detergent insoluble protein) were calculated as described by Licitra et al. [30]. The starch concentration was assed based on the AOAC method 920.40, the ether extract (EE) was quantified according to the AOAC method 920.39 using a Soxhlet apparatus (Velp SER 148/6, Velp Scientifica, Usmate, Italy). Ash-corrected, α-amylase-treated neutral detergent fiber (NDF) with addition of sodium sulfite (aNDFom), acid detergent fiber (ADF), and acid detergent lignin (ADL) were analyzed according to Mertens et al. [31] and AOAC 973.18.Undigested NDF (uNDF) was analyzed according to Cotanch et al. [32], and ash was analyzed according to AOAC (method 942.05). The starch content of WDGS was determined, while lipids were extracted with petroleum after hydrolysis with HCl, as described in Mammi et al. [23].

Feeds’ (CTR, FFP, and WDGS) fatty acid (FA) analysis was performed after lipid content extraction using a 2:1 *v*/*v* chloroform–methanol mixture, following the Folch method [33]. Fifteen milligrams of lipids were converted into FAME (fatty acid methyl ester) through acid-catalyzed transesterification, employing 2 mL of 0.5 M hydrochloric acid in methanol and 1 mL of hexane containing nonadecanoic acid (C19:0) at a concentration of 1 mg/mL as an internal standard (procured from Sigma-Aldrich Chemie; Taufkirchen, Germany).

Gas chromatographic analysis was conducted using a Shimadzu GC 2025 instrument (Shimadzu Corp.; Kyoto, Japan) equipped with a flame-ionization detector and a polar fused silica high-capillary column (SP-2650 FAME GC Column, 100 m, 0.25 mm i.d., 7” cage; supplied by Supelco Inc.; Bellefonte, PA, USA), with helium serving as the carrier gas at a flow rate of 30 mL/min. The comprehensive FAME profile in a 1 µL sample volume at a split ratio of 1:110 was determined under specific conditions: the oven temperature was initially set at 150 °C and maintained for 2 min, then ramped up to 220 °C at a rate of 1.5 °C/min and held for 20 min. The injector and detector temperatures were set at 250 °C. Identification of FAME compounds relied on a blend of a 37-component FAME mix (Supelco Inc.; Bellefonte, PA, USA). Feeds’ fatty acids were expressed in grams per 100 g of total lipids.

In order to determine total tract fiber digestibility (TTDpdNDF), a subsample of 6 cows per group (12 cows in total) was selected based on body weight (kg 628 ± 89), parity (n, 1.7 ± 0.9), stage of lactation (DIM, 125.1 ± 58.4), and milk production (kg/d, 44 ± 9.4). Fecal samples were collected from these cows during the experimental weeks for 2 consecutive days, 2 times a day, at 8 A.M. and 6 P.M. The fecal samples were dried in a forced-air oven at 65 °C for 48 h for DM determination and finely grinded with Cyclotec until they reached 1 mm particles size (FOSS Tecator; Hoganas, Sweden). Composition (moisture, starch, protein, lipids, ash, and fiber fractions) and fiber digestibility at 24 (pdNDF_24_) and 240 h (pdNDF_240_) were analyzed by NIRs to evaluate the total tract fiber degradability (TTDpdNDF) of diets [34]. The TTDpdNDF was calculated according to Palmonari et al. [35] using the following formula:

$$TTDpdNDF\left(\%pdNDF\right):100-\left(\left(\frac{uNDFdiet}{uNDFfeces}\right)\times \left(\frac{pdNDFfeces}{pdNDFdiet}\right)\right)\times 100,$$

where TTDpdNDF (% pdNDF) is the total tract digestibility of potentially digestible NDF, uNDF is the unavailable NDF, and pdNDF is the potentially digestible NDF.

### 2.3. Milking and Cheese Production

Milk obtained by each group was stored in separated and dedicated tanks. In the last week of each period, for two consecutive days (day 1 and day 2), a quote of milk obtained during the evening and morning milking of each group was collected and used for cheese production. The cheese was produced in the laboratories of the Department of Agricultural and Food Sciences of the University of Bologna, each equipped with 5 cheesemaking steel vats of 25 kg of capacity. During each experimental week, on day 1, 50 kg of milk was collected by group 1 and 75 kg by group 2. The opposite procedure was performed on day 2 in order to produce 5 cheeses per group/period, for a total of 40 cheeses (20/group). After collection, milk was maintained refrigerated (4 °C ± 1) until the beginning of the cheesemaking process. The amount of milk added and cooked in each vat was measured by a magnetic flowmeter (Danfoss MAGFLO Flowmeter, model MAG 6000, Danfoss, Nordborg, Denmark) and recorded, together with the vat number and the code of the cheese produced in that vat. Cheese production was performed always by the same operators following a specific protocol. In order to maintain consistency over time of the cheesemaking process, all relevant technological parameters (i.e., the dose of the starter, pH and °SH of milk, clot setting times, coagulation, and curd hardening times) were recorded (the data are shown in Supplementary Table S1). A detailed description of the cheesemaking process is shown in Table 5. The cheeses were marked with a colored casein plate, red or green, assigned at the beginning of the trial to one of the two treatments (TRT 1 or 2) without specification of the diet (CTR of WDGS + FF) in order to keep the cheesemakers blinded to the treatment. The cheeses were then stored together in the same ripening room (18 °C) for 3 months.

### 2.4. Milk and Cheese Analysis

Individual milk production and composition (fat, protein, and lactose content, measured in percentage) was recorded daily by the Afilab system (Afimilk; Afikim, Israel) in the milking parlor. Fat-corrected milk (FCM) and energy-corrected milk (ECM) were calculated according to Davidson et al. [36], using the following formulas:

ECM = [milk yield (kg/d) × 0.327] + [milk fat (kg/d) × 12.86] + [milk true protein (kg/d) × 7.65]

FCM = [milk yield (kg/d) × 0.4324] + [milk fat (kg/d) × 16.2162].

During the experimental weeks, tank milk, obtained by the evening and morning milking of each group, was sampled twice a week and analyzed by a qualified lab (Granlatte Granarolo spa; Bologna, Italy) for the content of fat, crude protein, total lactose, urea, total bacterial count (TBC), pH, titratable acidity (°SH/50 mL), and somatic cell count (SCC). In order to achieve normal distribution, the SCC data were transformed into somatic cell score (SCS) according to the Shook and Schutz method [37].

An aliquot of each sample was delivered to the official laboratories of Istituto Zooprofilattico Sperimentale della Lombardia e dell’Emilia-Romagna (IZSLER) to determine the milk coagulation properties using lactodynamographic (LDG) analysis, involving clotting time (r′) (time needed for the beginning of coagulation), curd firmness (a30) (time needed to reach 20 mm of amplitude on the chart), and curd firming time (k20) (amplitude of the chart, in mm). This analysis was performed with a Formagraph apparatus (Foss Eletric; Hillerød, Denmark) under isothermal conditions at 35 °C [38]. The evaluation of all LDG analyses permitted the evaluation of LDG type. LDG type indicates the rennet coagulation aptitude. Types A, B, and C have optimal and good coagulation aptitude, D types are characterized by short r′, short k20, and very high a30, while E and F relate to the clotting ability, with long and very long r′ and k20 and weak a30 [39].

The fatty acid profile of milk was determined by the Animal Production and Food Safety (SPASA) lab of the DIMEVET Department of the University of Bologna, whose laboratory is accredited according to UNI EN ISO 9001:2015 [28]. Fatty acids methyl esters were evaluated by capillary gas-chromatography after lipids extraction performed by the Feng method [40].

Shortly, 15 mL of milk samples were centrifuged in a plastic tube at 15,000× *g* for 30 min at 4 °C. The fat layer on top was removed and transferred in into a 2 mL Eppendorf and placed in a heater bath at 20 °C for 30 min. Following this, microtubes were centrifuged at room temperature for 20 min at 14,000 rpm using a Beckman Coaulter microfuge (Beckman Coaulter Inc., Brea, CA, USA). A 15 µL of top fat film was removed and transferred in amber vials and suspended in 1 mL nHexane. For internal standards and reference, we used Decanoic acid and Nonadecanoic acid. Transesterification for fatty acid methylester (FAME) preparation was carried out following the method described by Christie et al., 1982 [41]. Gas chromato-graphic analysis was conducted using Shimadzu GC2025 (Shimadzu, Kyoto, Japan), which was fitted with a flame ionization detector (FID) and a polar-fused silica capillary column (J&W Select FAME GC Column, Agilent, Santa Clara, CA, USA) 100 m, 0.25 mm, 7-inch cage). Helium was the carrier gas at a constant flow of 30 mL/min. The total FAME profile in a 1 µL sample volume at a split ratio of 1:80 was determined using the following GC conditions: the oven temperature was programmed at 40 °C and held for 1 min, then increased to 160 °C at 2 °C/min, held for 10 min, then increased up to 180 °C at 1.5 °C/min, held for 7 min, then increased up to 187 °C at 2 °C/min, held for 10 min, and then increased up to 220 °C at 3 °C/min, held for 25 min. The injector and detector were maintained at 270 and 300 °C, respectively. Identification of FAME was performed using a standard mixture of 37-component FAME Mix (Supelco; Bellafonte, PA, USA) and 20 individual FAME standards (Larodan Fine Chemicals; Malmo, Sweden). The isomers of 18:1 and 18:2 were analyzed using commercial standard mixtures (Larodan Fine Chemicals) and on chromatograms following Kramer et al. [42] and Alves and Bessa [43]. For each FA, response factors to FID and inter- and intra-assay coefficients of variation were calculated using a reference standard butter (CRM 164, Community Bureau of Reference; Brussels, Belgium). The fatty acids were expressed as g/100 g. Single fatty acids were grouped in de novo, mixed, and preformed following Woolpert et al. [44]. Briefly, de novo fatty acids were calculated as the sum of C4–C14 concentration, mixed fatty acids were the sum of C16, C16:1, and C17, and preformed fatty acids were the sum of ≥C18.

Cheese produced during the trial was weighed 24 h after production and at the end of the ripening period (3 months) to determine cheese yield (%), calculated as kg of cheese/kg of milk in the vat. After 3 months of maturation, one cheese per treatment (CTR or WDG + FFP) for each day of production (2 days per period) was randomly sampled among those produced on the same day and analyzed for composition and fatty acid profile (FA), while all cheeses produced were analyzed for organoleptic characteristics.

Analysis of cheese composition was performed by the Animal Production and Food Safety (SPASA) lab of the DIMEVET Department of the University of Bologna, whose laboratory is accredited according to UNI EN ISO 9001:2015 [28]. A quarter of each cheese was grinded, and two aliquots were collected to determine moisture (ISO 5534:2004) [45], fat (ISO 23319:2022) [46], protein content (ISO 8968-1:2014) [47], and FA. Lipid extraction was performed using the Folch method [33], while acid-catalyzed transmethylation was performed according to Stoffel et al. 1959 [48] in order to recover the free fatty acid component of ripened cheese as well [49]. The fatty acids methyl esters were evaluated as previously described for milk samples [50].

Sensory analysis of all cheese produced (20/group) was performed by CRPA (Research Center for Animal Production; Reggio Emilia, Italy), applying a quantitative descriptive analysis test (QDA) to determine the complete sensory profile of cheese, considering view, olfaction, taste, aftertaste, and structure. The evaluation was performed by 7 selected and trained panelists according to EN ISO 13299 [51]. Each panelist evaluated two replicates of each sample served at a fixed temperature of 16 ± 2 °C following a blind random order. The sensory profile was determined according to the ETANA model described by Bozzetti et al. [52]. According to that model, each of the 14 features, shown in Table 6, was evaluated using a graduated scale from 1 (absence of sensation) to 7 (highest intensity of sensation).

### 2.5. Environmental Impact

The environmental impact of the two diets was calculated by an LCA (life cycle assessment) study conducted by Life Cycle Engineering S.p.a. (Via Livorno 60, Environment Park, Turin, Italy) following a “from cradle to farm gate” LCA procedure. Considering that the two diets differed only in the partial substitution of traditional starch and protein source with WDGS + FFP, the impact of this substitution was evaluated, including the different stages of production. Enteric fermentation, waste management, and processes associated with farming and milk transformation were not included in the LCA calculation because they are common in both diets. The indicators evaluated were global warming potential (GWP, kg CO_2_ eq), net fresh water requirements (H_2_O, kg), and land occupation, calculated as area × time (m^2^a), per kg of diet, and per kg of milk produced [53,54]. In this work, the economic allocation of factors was adopted considering resource utilization, cost-effectiveness, and distribution efficiency. Hay produced by the farm was assumed to be transported for 20 km, while for other feeds, as suggested by Bragaglio et al. [55], the distance from the farm was calculated considering 200 km for traditional feeds and 300 km for the circular ones (WDGS and FFP) considering the distance of the farm from the plant production.

### 2.6. Statistical Analysis

Statistical analysis was performed using the software JMP Pro (v15, Statistical Analysis Systems Institute Inc.; Cary, NC, USA).

The research was designed as a double cross-over trial with two dietary treatments (CTR or WDGS + FFP) as the main effect. As previously described, the treatments were administered alternatively to two groups for 4 experimental periods, each group receiving the treatments twice. The periods were composed of 4 weeks of adaptation and 1 week of data collection.

Normal distribution of data was tested using the Shapiro–Wilk test and, accordingly, the data were analyzed with different linear mixed models with diet (CTR or WDGS + FFP) as a fixed effect. The group served as an experimental unit and it was, therefore, included in the model as a random effect, along with the period (1, 2, 3, 4). When a significant F test (*p*  < 0.05) was detected, means’ multiple comparison was performed by the Tukey–Kramer’s test.

## 3. Results

### 3.1. Animal Performance

Results about the dry matter intake, milk yield, rumination time, and fiber digestibility are reported in Table 7. Briefly, dietary treatment had no influence on the group’s average DMI (CTR = 22.3 kg/d; WDGS + FFP = 22.92 kg/d, *p* = 0.14), milk yield (CTR = 32.45 kg/d; WDGS + FFP = 32.70 kg/d, *p* = 0.27), and total tract fiber digestibility (CTR = 80.52; WDGS + FFP = 82.08, TTDpdNDF, %pdNDF, *p* = 0.70). The daily time spent ruminating changed significantly with the different diets (CTR = 499.49 min/d; WDGS + FFP = 527.10 min/d; *p* < 0.0001), but it always remained around physiological levels in both groups.

### 3.2. Milk Composition

Milk composition is reported in Table 8.

Milk analysis revealed some significant variations between treatments. Fat content was significantly lower in the WDGS + FFP treatment compared to the CTR, with an average percentage of 3.42% compared to 3.71%, respectively (*p*-value = 0.03). On the contrary, no significant differences were observed in protein, lactose, and urea levels between the two treatments (*p*-value > 0.05). The mean values of milk protein and lactose were 3.33% and 4.81% (CTR), and 3.31% and 4.82% for the experimental group (WDGS + FFP). Similarly, no significant differences were observed for total bacterial count (TBC), somatic cell score (SCS), and milk coagulation properties evaluated by lactodynamographic analysis (*p*-value > 0.05). Indeed, all samples fell into category A and, therefore, were classified with an optimal coagulation aptitude (CTR = 100% LDG, type A and WDGS + FFP = 100% LDG, type A).

#### Milk Fatty Acid Profile

As reported in Table 9, the analysis of fatty acid composition in milk fat revealed few significant variations between treatments in odd- and branched-chain fatty acids (OBCFA), which were present in higher concentrations in CTR (3.46% FA) compared to WDGS + FFP (3.29% FA) (*p*-value 0.048).

A significant reduction was also observed on some medium-chain SFAs in the WDGS + FFP milk, as well as a tendency for a reduction in de novo FA (20.62 CTR vs. 19.85 WDGS + FFP, *p*-value = 0.08). On the contrary, the total amount of SFA, monounsaturated (MUFA), and polyunsaturated (PUFA) FA, as well as the mixed and preformed groups, were not influenced by the treatment (*p*-value > 0.05).

Similarly, the percentages of n-3 and n-6 fatty acids, as well as the n-6: n-3 ratio, did not exhibit significant differences between CTR and WDGS + FFP, indicating a similar balance of these essential fatty acids in both treatments (*p*-value > 0.05). The same absence of influence was observed considering the percentage of conjugated linoleic acid (CLA) (*p*-value = 0.24).

The data are reported in detail in Table 9.

### 3.3. Cheese Composition and Sensory Analysis

Cheese characteristics produced by control and treated milk were investigated; the results are detailed in Table 10.

The weights of cheeses 24 h post-production were similar: 2.16 kg and 2.08 kg for treated and control milk, respectively (*p* > 0.05). T cheese yields at 24 h for treated milk (9.82%) and for the control (9.48%) (*p* > 0.05) were also similar. After 3 months of ripening, the cheeses showed the same similarity between weight (1.75 vs. 1.76 kg for CTR and WDGS + FFP, respectively, *p* > 0.05) and cheese yield (7.95% and 8.01% for CTR and WDGS + FFP, respectively, *p* > 0.05), confirming the absence of impact on cheese yield of the circular feed.

The same results were observed considering the chemical composition of cheese, which is reported in Table 10. DM, moisture, and protein content were very similar between diets, while a tendency for a slight reduction in fat content emerged in circular cheese compared to the control (CTR = 32.85% DM, WDGS + FFP = 31.88% DM, *p*-value = 0.06). Interestingly, the fatty acid profile of cheese fat, expressed as a percentage of total fatty acids, varied between diets, as detailed in Table 11, with notable differences in saturated and monounsaturated FAs.

**Figure 1:**
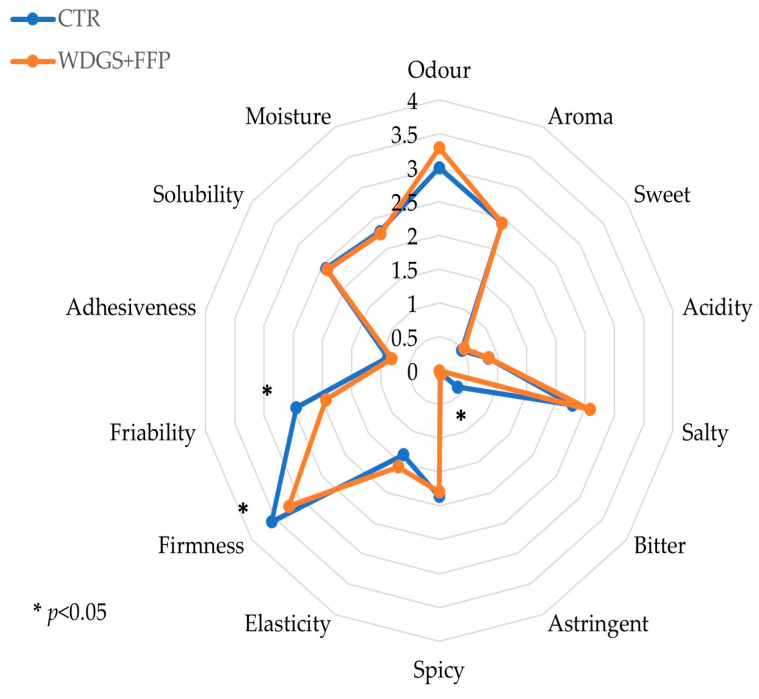
Sensory profile of cheese produced by treated and control groups evaluated using a quantitative descriptive analysis test and performed by a trained expert panel. CTR: Control; WDGS + FFP: Condensed wheat distiller soluble + former foodstuff.

Significant differences were observed in saturated fatty acid content, with the CTR treatment showing a higher proportion (69.27% of tot. FA) compared to WDGS + FF (66.79% of total FA) (*p*-value = 0.0018). Monounsaturated fatty acids were higher in the WDGS + FFP treatment (28.90% of total FA) compared to the control (26.47% of total FA) (*p*-value = 0.0007). Particularly, the WDGS + FF cheese had a significant (*p* < 0.05) reduction in C14:0 iso, C15:0 iso, and C16:0 iso in comparison with the CTR and an increase (*p*-value < 0.05) in the oleic acid (C18:1 c9): 19.50% vs. 21.10% of total FA, in the CTR and WDGS + FFP group, respectively (Table 11). On the contrary, polyunsaturated fatty acids showed no significant difference between treatments, with both CTR and WDGS + FFP treatments displaying similar proportions (CTR = 4.07% vs. WDGS + FFP= 4.06% of total FA; *p*-value < 0.5). The same results were observed when considering n-3 and n-6 fatty acids as well as odd- and branched-chain FA. Referring to the classification that considered the synthesis pathways [44], no differences were observed between treatments for de novo and mixed FAs, while a significant difference was detected in the preformed ones with 34.22% in CTR vs. 37.04% of total FA in WDGS + FFP (*p*-value = 0.0041). The sensory descriptors of cheese, evaluated through a quantitative descriptive analysis test, revealed a comparable profile of cheeses with only a few significant differences between the treated (WDGS + FFP) and control (CTR) groups. For a comprehensive understanding of the sensory profile, refer to Figure 1, which provides a visual representation of the complete sensory profile evaluated by the expert panel. The few differences regard bitterness, which appeared to be completely absent in the circular cheese (0.00 vs. 0.39 intensity for the WDGS + FFP and CTR, respectively, *p*-value < 0.05), firmness, (3.21 for WDGS + FFP vs. 3.58 for CTR; *p*-value < 0.01), and friability: 1.94 vs. 2.45 for WDGS + FFP and CTR, respectively (*p*-value < 0.05).

### 3.4. Environmental Impact

The results obtained by the LCA analysis showed an important reduction in the overall environmental impact of the “circular” diet compared to the traditional one. All the 3 indicators, land occupation (m^2^a), global warming potential (kg CO_2_ eq.), and net fresh water (kg) were lower for the WDGS + FFP diet when calculated for both kg of diet and kg of milk produced (Table 12). In particular, for each kg of the “circular” diet, land occupation decreased by 24%, the GWP by 25% and the net fresh water decreased by 31%. Similar results were obtained when the impact of the diet was calculated per kg of milk produced: land occupation decreased by 19%, the GWP by 20% and net fresh water by 26%.

## 4. Discussion

The aim of this study was to investigate the effects of including agro-industrial by-products and bakery former foodstuffs in the diet of dairy cows on milk and cheese quality. Most of the studies on alternative ingredients for cow rations considered nutritional content, palatability, and digestibility [9] among other physiological aspects, such as ruminal activity and pH [23]. These studies reported encouraging results on the possibility of including these ingredients in cow diets without negative effects on rumen environment, fiber digestibility, and milk production [23].

However, to the best of our knowledge, few studies have investigated the influence of these “circular” feeds on the composition and quality of the milk and the final characteristics of the cheese produced.

Among the different studies, the composition of WDGS shows significant variability due to the raw materials used [57]. The WDGS used in our study had a lower crude protein content (23.53%), higher starch (17.14%), and lower aNDFom (1.1%) than those reported by Duncan et al. [58] and Buenavista et al. [59], who reported that the level of CP, starch, and aNDFom were between 31.8 and 36.7; 3.2, and 24.2–41.4, respectively.

Similarly, the composition of FFP varies widely, as it is being influenced by several factors, such as the type of raw materials, origin, and processing methods. The FFP used in our study had similar nutritional characteristics to those reported in literature [15]. In particular, the FFP are characterized by a considerable amount of starch with high degradability due to its ingredients mainly consisting cooked starch sources such as biscuits, pasta, and bread [15]. These aspects suggest that the inclusion of FFP in ruminant diets could have a positive effect on fiber and starch digestibility and overall feed efficiency. In addition, in our study, FFP had higher levels of SFA and MUFA compared to cereal-based feeds used in traditional dairy cow diets. This trend is consistent with the observations of Humer et al. [60], who noted that bakery by-products (BP) are rich in saturated fatty acids (SFA) and monounsaturated fatty acids (MUFA), especially C18:1 n9, suggesting a potential impact on rumen health and the fatty acid profile of milk. The first aspect was previously explored by Mammi et al. [23], who reported no effect on rumen pH and milk composition with a limited amount of BP inclusion.

Animal performance was not the main focus of this study, considering that these aspects have been previously investigated [18,21,23]. However, the inclusion of WDGS and FFP in the diet did not significantly affect average group dry matter intake, milk yield, or total tract fiber digestibility; this is consistent with the results of our previous study focusing on animal performance and rumen health [23]. The addition of WDGS and FFP had a positive effect on rumination time, increasing it to 527.10 min per day (*p*-value < 0.001), excluding the adverse effects on cows’ digestive health. A part of these results could be related to the higher humidity of the circular PMR (WDGS + FFP) due to the inclusion of WDGS. In this study, the evolution of particle size in PMR over the day was not measured, but rumination time is strongly related to the forages’ consumption [61,62], and the WDGS could have reduced the sorting activity by cows in PMR. This observation is consistent with the results reported by Kaltenegger et al. [3], who found that the inclusion of bakery by-products led to increased rumination.

Regarding milk composition, our results only partially agree with other studies. Interestingly, while the inclusion of WDGS and FFP did not significantly affect most of the milk components, a reduction in milk fat was observed (3.71% CTR vs. 3.42% WDGS + FF). This finding contrasts with Kaltenegger et al. [3], who reported an increase in milk fat and a decrease in protein content as a result of the addition of bakery by-products to the diet. However, Kaltenegger’s study used bakery by-products alone, whereas our study combined WDGS and FFP, which may partly explain the different results. In our study, the starch and aNDFom contents of the two diets were comparable, as well as the peNDFom, so the difference in milk fat content could be related to the fatty acid composition of the “circular” diet, which was particularly rich in both MUFA and PUFA. However, the fatty acid profile of ruminant products does not directly reflect the one in dietary sources due to the biohydrogenation of fatty acids in the rumen. This process is strictly dependent on the rumen’s environment and pH, and enables the conversion of a significant percentage of dietary MUFA and PUFA into SFA, particularly C18:0, which is then absorbed and metabolized by the mammary gland [63,64]. Consequently, the availability of dietary unsaturated fatty acids for direct incorporation into milk fat is limited, leading to a relatively stable milk fatty acid profile despite dietary modifications [65].

In our study, the inclusion of WDGS and FFP did not significantly alter the total proportions of SFA and MUFA, which remained similar between the two diets. However, we observed a reduction in the concentration of some medium-chain SFAs (C10–C15) and in odd- and branched-chain fatty acids (OBCFAs). OBCFAs are mainly produced by cellulolytic bacteria in the rumen, and their presence in milk is reduced by high dietary FA [66]. These results could, therefore, indicate a possible interaction of FFP with the rumen microbial population [67].

In their study, Khiaosa-ard et al. [67] found a significant reduction in SFA, especially C16:0, and a significant increase in unsaturated fatty acids, mainly represented by C18:1 cis-9. In our study, the concentration of these FAs in milk was not affected by the treatment, contrary to what we observed in cheese. However, the general trend remained the same in both products, with MUFA increasing, SFA decreasing, and PUFA remaining stable between treatments.

Indeed, after 3 months of ripening, we observed a decrease in the cheese content of C16:0 and total SFA and an increase in MUFA, mainly represented by C18:1 cis 9 (CTR = 19.50, WDGS + FFP = 21.10 g/100 g FA, *p* value = 0.028). Although with different types of diets, previous studies have shown that the inclusion in the diet of by-products rich in MUFA, such as olive pomace, has a significant effect on the fatty acid profile of cheese, characterized by an increase in MUFA and a reduction in SFA [68,69]. These changes reflect the FA composition of the diet and support the hypothesis of positively influencing the fat profile of cheese by reducing SFA in favor of MUFA, which are considered beneficial for human health [70,71,72]. In addition to dietary effects, cheese ripening processes, including lipolysis and microbial activity, further contribute to changes in the fatty acid profile. Lipolysis, which occurs during ripening, is driven by milk lipases and microbial enzymes that hydrolyse triglycerides, leading to the release of free fatty acids [69]. This process predominantly affects short- and medium-chain fatty acids (C4:0–C12:0), while microbial and rennet-derived lipases are more active on long-chain fatty acids (C14:0–C18:1) [73]. The combined effect of diet and enzymatic activity should explain the change in FA profiles. It was more evident in cheese than in milk, where the concentration of these fatty acids remained relatively stable.

PUFA concentration was not affected in our study, neither in milk nor in cheese fat, in agreement with the results obtained by Khiaosa-ard [67], who tested bakery BP at 15% and 30% DMI inclusion levels and observed no significant changes in the proportions of milk PUFA levels. Dietary PUFAs are significantly reduced by ruminal biohydrogenation, the efficiency of which is strictly dependent on the rumen environment, in particular pH and the abundance of microflora [74]. These results suggest that these levels of inclusion of bakery by-products have no negative effects on the rumen environment, as also reported in previous works [23].

Coagulation aptitude of milk, which is fundamental in determining the final cheese production [75], was assessed using LDG analysis and resulted to be optimal for both diets. This result explains the absence of the difference between the final yield and the composition of the cheese after 3 months of ripening. Similarly, the sensory profile of the ripened cheese was not affected either, despite the differences observed in SFA and MUFA contents. Only a few descriptors, such as bitterness, hardness, and crumbliness, were more pronounced in the control group. This result could be explained by the fact that in our study, the main differences in cheese FAs were related to long-chain FAs. These compounds are less involved in the sensory properties of cheese, which is mainly driven by short-chain FAs [76].

Indeed, the composition of milk fat is essential for the sensory profile of cheese due to the volatile compounds produced by enzymatic and bacterial activities during the ripening process [77,78]. This aspect is particularly important when considering typical and PDO productions, which are strictly dependent on their qualitative and sensorial characteristics.

Another aspect examined in our study is the environmental impact of the two diets in terms of land and water use and GWP. This factor is particularly crucial for livestock production. Recent studies have estimated that feed crop production accounts for 33% of global arable land, suggesting that even modest improvements in diet composition could substantially reduce the need for further land expansion for feed production. [79,80]. In our study, the results of the LCA analysis showed that incorporating by-products and former food in cow rations reduces both land and water requirements for feed production as well as the overall global warming potential of the animals’ diet.

## 5. Conclusions

The results of the present study evidenced that the inclusion of WDGS and FFP in the diet of dairy cows, in a defined amount, did not adversely affect animal productivity or milk composition, but it brought important and significant benefits in terms of sustainability of dairy farming. Indeed, the substitution of conventional feeds with these by-products significantly reduced the environmental impact of cows’ diets by limiting land use and net blue water consumption for crop farming and overall reducing greenhouse gas emissions of the cheese production. Moreover, the inclusion of these by-products did not significantly change milk and cheese yield. Interestingly, some differences were found in milk fat content and in the fatty acid profiles of milk and cheese, particularly on MUFA and SFA content. This aspect needs to be considered during feed and diet formulation. However, the increase in MUFA and the reduction in SFA content of cheese could be considered as potential improvements in the nutritional value of milk and cheese fat, bearing in mind the beneficial effects of MUFA on human health. Despite this, the cheese organoleptic profile was not affected by the diets, except for some negligible changes in a few sensory parameters. Ultimately, this study provided promising evidence of the safety of including these by-products in a balanced dairy cow diet at a defined level, in terms of milk and cheese quality. Strong evidence emerges from the LCA results, demonstrating that the use of these circular feeds represents a viable and sustainable food strategy for the dairy sector. However, wider studies focusing on particular systems (e.g., PDO cheese) should confirm the impact of these feeds on the quality of the final products and explore the economic and environmental large-scale impacts of this approach that could contribute to a more efficient management of feed resources in the dairy chain.

## Figures and Tables

**Table 1:** Sequence of dietary treatments administration during the trial.

	Dietary Treatments ^1^	
Experimental Periods ^2^	Group 1	Group 2
1	CTR	WDGS + FFP
2	WDGS + FFP	CTR
3	CTR	WDGS + FFP
4	WDGS + FFP	CTRT

^1^ CTR: control; WDGS + FFP: condensed wheat distiller soluble + former foodstuff. ^2^ Experimental period: 4 weeks of adaptation and 1 week of data recording and cheese production.

**Table 2:** Ingredients and chemical composition of the dietary treatments ^1^.

Ingredients, kg/d as Fed	CTR ^1^	WDGS + FFP ^1^
Alfalfa hay	7.0	7.0
Grass hay	3.0	3.0
Wheat Straw	1.5	1.5
Cereal mix flakes ^2^	6.0	4.7
Lactation mix CTR ^3^	10.0	6.7
Megafat^® 4^	0.5	0.5
Former foodstuff feed (FFP) ^5^	--	3.0
Wheat wet distiller soluble (WDGS) ^6^	--	4.0
Self-feeder pellet ^7^	3.0	3.0
Total, as fed	31.0	33.4
**Composition of PMR ^8^, % DM ^9^**		
Samples, n	23	23
DM, % as fed	88.5 ± 2.3	82.8 ± 2.7
Moisture	11.5 ± 2.3	17.2 ± 2.7
CP	14.2 ± 1.1	14.1 ± 1.2
Soluble CP	4.39 ± 1.6	14.32 ± 1.5
NDIP	3.31 ± 0.9	3.21 ± 1.0
ADIP	0.87 ± 0.6	0.84 ± 0.9
Starch	21.5 ± 4.1	21.7 ± 2.7
EE	3.4 ± 0.2	3.7 ± 0.3
ANDFom	37.8 ± 3.3	37.9 ± 2.9
ADF	26.7 ± 1.9	25.8 ± 1.3
ADL	5.3 ± 0.3	5.2 ± 0.3
uNDF_240_	13.2 ± 1.8	12.7 ± 1.3
Ash	6.8 ± 0.6	7.1 ± 0.7
PeNDFom	16.7 ± 1.3	18.2 ± 2
Metabolizable energy (Mcal/kg)	2.60	2.62

^1^ CTR: control; WDGS + FFP: condensed wheat distiller soluble + former foodstuff. ^2^ Cereal mix flakes: 50% corn flakes, 50% sorghum flakes. ^3^ Lactation mix CTR (Preti Mangimi Srl, Borgo Mantovano, Italy): 34.6% wheat bran, 24.4% corn gluten meal, 18.4% soy hulls, 6.1% soybean meal. 4.8% propylene glycol, 4.1% molasses, 4% corn, 1.5% baking soda, 1.3% calcium carbonate, 1.2% monocalcium phosphate, 1.2% sodium chloride, 1.17% calcium sulfate; 1.1% brewer’s yeast; 0.45% magnesium oxide, 0.15% Vitamin supplements A, D3, E, 0.2% trace elements, 0.13% Smartamina M. ^4^ Megafat® (Volac Socoor srl; Milano, Italy): Fat prill, 99%% Fat with 88%Total Fatty Acids C16:0, 8% Total Fatty Acids C18:1. ^5^ Former foodstuff feed (Dalma spa; Marene, Italy). Composed of bakery industry wastes like pasta, bread, biscuits, and snacks no longer intended for human consumption. Analysis reported in Table 3. ^6^ Wheat wet distiller soluble (Dalma spa; Marene, Italy). Analysis reported in Table 3. ^7^ Self-feeder pellets: 18.4% corn, 18.4% sorghum, 12.0% wheat bran, 12.0% beet pulp, 12.0% soy hulls, 8.0% whole soy flakes, 8.0% soy extraction flour 48% PG, 5.0% carob, 5.0% molasses, 0.95% sodium chloride, 0.15% vitamin supplements A, D3, E, 0.2% trace elements. ^8^ Partial mixed ration (Ration without self-feeder pellet). ^9^ DM: Dry matter; CP: Crude protein; NDIP: Neutral detergent insoluble protein, ADIP: Acid detergent insoluble protien, aNDFom: Amylase-treated ash-corrected neutral detergent fiber (NDF) with addition of sodium sulphite; ADF: Acid detergent fiber; ADL: Acid detergent lignin; uNDF: Undigested NDF at 240-h in vitro fermentation; peNDFom: Physically effective ash-corrected neutral detergent fiber (NDF) with addition of sodium sulphite.

**Table 3:** Chemical composition ^1^ (% DM) of the different preparations tested ^2^.

Component	Self-Feeder Pellet	CTR ^2^	FFP ^2^	WDGS ^2^
DM, % as feed	88.7 ± 0.3	89.25 ± 1.15	90.57 ± 1.05	36.00 ± 0.81
CP	16.60 ± 1.0	16.00 ± 0.57	11.88 ± 0.58	23.52 ± 0.93
EE	4.20 ± 0.52	3.25 ± 0.31	6.00 ± 0.5	4.29 ± 0.32
Starch	29.14 ± 2.17	29.02 ± 1.56	41.33 ± 2.03	17.14 ± 1.15
aNDFom	23.07 ± 1.25	23.75 ± 1.42	21.54 ± 1.30	1.1 ± 0.12
Starch digestibility (7 h), %	62.00 ± 1.90	59.7 ± 2.5	93.03 ± 2.03	-

^1^ DM: Dry matter; CP: Crude protein; EE: Ether extract; aNDFom: Amylase-treated ash-corrected NDF with addition of sodium sulphite; ^2^ CTR: Lactation mix; WDGS: Condensed wheat distiller soluble; FFP: Former foodstuff.

**Table 4:** Fatty acids composition of feeds (CTR, FFP, and WDGS) ^1^ and total fatty acids in the diets derived from the experimental preparations tested (CTR and WDGS + FFP) ^2^.

	Feed			Diet	
Fatty Acids (g/100 g Total Lipids)	CTR ^1^	FFP ^1^	WDGS ^1^	CTR ^2^	WDGS + FFP ^2^
Total Fatty acids	69.57	91.74	63.59	226.09	425.73
C12:0	0.01	0.57	0.01	0.04	1.06
C14:0	0.16	0.68	0.11	0.52	1.77
C15:0	0.05	0.06	0.08	0.18	0.37
C16:0	11.04	14.25	15.51	35.89	76.31
C16:1c7	0.10	0.05	0.06	0.33	0.41
C16:1c9	0.25	0.32	0.13	0.82	1.34
C17:0	0.08	0.10	0.09	0.26	0.51
C17:1c10	0.04	0.04	0.03	0.12	0.20
C18:0	2.06	7.98	0.99	6.70	20.55
C18:1c9	15.27	44.89	7.55	49.63	127.01
C18:1c11	0.84	0.90	0.70	2.73	4.64
C18:2cc	35.60	19.17	35.08	115.71	172.24
C20:0	0.18	0.38	0.09	0.59	1.23
C20:1c11	0.41	0.26	0.38	1.33	2.01
C18:3n3	2.73	1.32	2.02	8.88	11.80
C20:2n6	0.08	0.04	0.06	0.27	0.37
C22:0	0.16	0.32	0.13	0.53	1.16
C22:1c13	0.05	0.03	0.06	0.17	0.27
C20:4n6	0.05	0.04	0.03	0.15	0.22
**Group of fatty acids**	**CTR ^1^**	**FFP ^1^**	**WDGS ^1^**	**CTR ^2^**	**WDGS + FFP ^2^**
SFA ^3^	13.95	24.59	17.19	45.35	104.13
UFA ^4^	55.61	67.15	46.40	180.74	321.60
PUFA ^5^	38.48	20.61	37.35	125.07	184.98
MUFA ^6^	17.13	46.55	9.06	55.66	136.62
PUFA n6	35.73	19.25	35.18	116.13	172.83
PUFA n3	2.75	1.32	2.08	8.95	11.94

^1^ CTR: Lactation mix; WDGS: Condensed wheat distiller soluble; FFP: Former foodstuff. ^2^ CTR: Control diet; WDGS + FFP: Experimental diet. ^3^ SFA: Unsaturated fatty acids. ^4^ UFA: Unsaturated fatty acids. ^5^ PUFA: Polyunsaturated fatty acids. ^6^ MUFA: Monounsaturated fatty acids.

**Table 5:** Description of the cheesemaking process.

Step	Description
Weighing of Raw Milk	In total, 22 kg of raw milk was weighed from the transport containers and introduced into the processing vat. Subsequently, milk temperature was measured, and the heating process commenced.
Heating Process	Controlled heating was applied, raising the milk temperature to 35–36 °C with continuous agitation. The pH levels were monitored upon reaching the desired temperature.
Addition of Commercial Starters	Lyophilized commercial starters (SACCO, *Streptococcus thermophilus* LYOFAST Ste, *Lactobacillus helveticus* LYOFAST LH in a 1:1 ratio) were reactivated in sterile saline solution (9 g/L NaCl). The reactivated starters were added to the vat and mixed with milk.
Resting Period	The milk was allowed to rest for approximately ten minutes, providing time for added microorganisms to adapt and initiate microbial activity.
Addition of Rennet	In total, 7.3 g of calf rennet powder with a concentration of 1:130,000 per liter of milk was added to initiate coagulation.
Coagulation	A resting period of approximately 17 min allowed the rennet to initiate coagulation. The milk was then left to rest for an additional 2–3 min until the curd set, which was determined empirically.
Curd cutting and processing	Coarse breaking of the curd was performed initially to facilitate whey drainage without causing excessive damage. Coarse breaking of the curd was performed approximately three minutes after the initial break. The curd was manipulated to achieve small granules, contributing to the desired texture. This granulated curd was then processed further to enhance its granular consistency.
Cooking Process	The cooking involved slow heating with continuous curd movement until reaching 43–44 °C, facilitating whey drainage. The temperature gradually increased to 53–54 °C, optimizing curd texture.
Curd Collection and shaping	The curd was collected within a linen cloth and maintained under whey for 20–30 min. It was then extracted, still within the cloth, and placed in specific plastic molds for shaping.
Pressing and Curing	The forms were placed in a thermostat at 45 °C for 4 h, reaching a pH of 5.20–5.30. The forms were turned every two hours and subjected to pressing with a 3 kg weight for the initial two hours.
Salting	The forms were immersed in a saturated brine solution.
Aging	The forms were placed in designated aging rooms and maintained for approximately 3 months at 18 °C.

**Table 6:** Cheese sensorial descriptors evaluated during a quantitative descriptive analysis test performed by a trained expert panel on control and treatment cheese samples.

Descriptor	Attribute
Smell	Total intensity of aroma and odor
Taste	Sweet, salted, bitter, spicy, acidity, astringent
Texture	Elasticity, firmness, friability, adhesiveness, solubility, moisture

**Table 7:** Effects of dietary treatments ^1^ on DMI ^2^, milk yield, rumination time, and fiber digestibility ^3^.

Item	CTR ^1^	WDGS + FFP ^1^	SEM	*p*-Value
DMI ^2^, kg/d	22.30	22.92	0.30	0.14
Milk, kg	32.45	32.70	0.22	0.27
Rumination, min/d.	499.49 ^a^	527.10 ^b^	6.29	<0.001
TTDpdNDF, %pdNDF ^3^	80.58	82.08	3.46	0.70

^a,b^ Values within a row with different superscripts differ (*p ≤* 0.05). ^1^ CTR: control; WDGS + FFP: condensed wheat distiller soluble + former foodstuff. ^2^ DMI: Dry matter intake. ^3^ TTDpdNDF, % pdNDF: Total tract digestibility of pdNDF.

**Table 8:** Effects of dietary treatments ^1^ on milk production, composition, and quality.

Item	CTR ^1^	WDGS + FFP ^1^	SEM	*p*-Value
ECM ^2^, kg	34.34	33.52	0.33	0.15
FCM 4% ^3^, kg	33.53	32.43	0.48	0.19
Fat, %	3.71 ^a^	3.42 ^b^	0.09	0.03
Protein, %	3.33	3.31	0.02	0.40
Lactose, %	4.81	4.82	0.003	0.78
Urea, g/100 g	19.77	16.42	1.90	0.24
SCC ^4^, cell × 1000/mL	352.83	290.33	76.13	0.57
SCS ^5^, points	4.56	4.41	0.33	0.75
LDG ^6^, type A, % samples	100	100	-	-
TBC ^7^, 1000 × cfu/mL	3.83	3.33	0.41	0.4

^a,b^ Value within a row with different superscripts differ (*p* ≤ 0.05). ^1^ CTR: control; WDGS + FFP: condensed wheat distiller soluble + former foodstuff. ^2^ Energy-corrected milk calculated according to Davidson et al. [36]. ^3^ Fat-corrected milk calculated for 4% fat content according to Davidson et al. [36]. ^4^ SCC: Somatic Cell Count. ^5^ SCS: Somatic cell score according to the Shook and Schutz method [37]. ^6^ LDG type A: Clotting time, evaluated using lactodynamographic analysis. The classification is according to Zannoni and Annibaldi [56]. ^7^ TBC: Total bacterial count.

**Table 9:** Fatty acid profile of bulk milk fat produced with the two dietary treatments ^1^.

Fatty Acids (g/100 g FA)	CTR ^1^	WDGS + FFP ^1^	SEM	*p*-Value
C4:0	2.26	2.25	0.030	0.75
C6:0	1.36	1.32	0.026	0.26
C8:0	0.78	0.74	0.017	0.12
C10:0	1.76	1.64	0.039	0.06
C10:1 c9	0.16	0.16	0.006	0.44
C11:0	0.05 ^a^	0.04 ^b^	0.002	0.02
C12:0	1.99 ^a^	1.86 ^b^	0.042	0.04
C12:1 c11	0.04	0.04	0.002	0.15
C13:0	0.10 ^a^	0.08 ^b^	0.003	0.01
C14:0 iso	0.06	0.07	0.004	0.74
C14:0	9.42	9.07	0.128	0.06
C15:0 iso	0.15	0.15	0.009	0.81
C15:0 ante	0.45	0.44	0.013	0.64
C14:1 c9	0.93	0.96	0.017	0.14
C15:0	1.10 ^a^	1.03 ^b^	0.018	0.02
C16:0 iso	0.19	0.19	0.006	0.61
C16:0	36.57	37.87	0.755	0.24
C16:1 t8	0.03 ^a^	0.04 ^b^	0.003	0.03
C16:1 t9	0.03 ^a^	0.04 ^b^	0.001	0.01
C17:0 iso	0.30	0.30	0.008	0.86
C16:1 c7	0.20 ^a^	0.21 ^b^	0.004	0.02
C16:1 c9	2.01 ^a^	2.22 ^b^	0.039	0.002
C17:0 ante	0.56	0.53	0.016	0.15
C17:0	0.50 ^a^	0.46 ^b^	0.007	0.005
C17:1 c9	0.25	0.25	0.005	0.52
C17:1 c10	0.03 ^a^	0.02 ^b^	0.002	0.009
C18:0	6.86	6.39	0.224	0.16
C18:1 t6-8	0.33	0.32	0.027	0.80
C18:1 t9	0.25	0.24	0.018	0.85
C18:1 t10	0.69	0.71	0.067	0.88
C18:1 t11	0.70	0.72	0.034	0.71
C18:1 c6	0.52	0.53	0.023	0.87
C18:1 c9	22.76	22.62	0.414	0.81
C18:1 t15	0.20	0.19	0.011	0.34
C18:1 c11	0.71	0.67	0.020	0.19
C18:1 c12	0.35	0.34	0.020	0.75
C18:1 c13	0.10	0.09	0.006	0.39
C18:1 t16	0.26	0.24	0.009	0.15
C18:2 t9t12	0.26	0.26	0.012	0.76
C18:2 cc	2.98	2.98	0.058	0.99
C20:0	0.07	0.07	0.002	0.96
C18:3n3	0.50	0.50	0.011	0.52
CLA9:11ct	0.48	0.51	0.019	0.24
CLA10:12tc	0.007	0.004	0.0014	0.15
C20:2n6	0.05	0.05	0.004	0.87
C22:0	0.04	0.04	0.003	0.11
C20:3n3	0.15	0.15	0.002	0.53
C20:4n6	0.19 ^a^	0.18 ^b^	0.003	0.05
C22:2	0.02	0.02	0.001	0.26
C24:0	0.03	0.03	0.002	0.33
**Groups of Fatty acids (g/100 g FA)**	**CTR ^1^**	**WDGS + FFP ^1^**	**SEM**	***p*-value**
OBCFA ^2^	3.46 ^a^	3.29 ^b^	0.056	0.05
SFA ^3^	64.61	64.57	0.624	0.96
MUFA ^4^	30.56	30.60	0.601	0.96
PUFA ^5^	4.63	4.65	0.062	0.86
n-3	3.29	3.30	0.012	0.48
n-6	0.66	0.64	0.062	0.94
n-6/n-3 ratio	5.03	5.15	0.168	0.62
CLA ^6^	0.48	0.51	0.021	0.24
De novo ^7^	20.62	19.85	0.29	0.08
Mixed ^7^	40.68	42.14	0.742	0.15
Preformed ^7^	38.51	37.84	0.771	0.54

^a,b^ Values within a row with different superscripts differ (*p* ≤ 0.05). ^1^ CTR: control; WDGS + FFP: condensed wheat distiller soluble + former foodstuff. ^2^ OBCFA: The sum of odd- and branched-chain fatty acids. ^3^ SFA: Short-chain fatty acids. ^4^ MUFA: Monounsaturated fatty acids. ^5^ PUFA: Polyunsaturated fatty acid. ^6^ CLA: Conjugated Linoleic Acid. ^7^ De novo (from C4 to C14); mixed (C16. C16:1. C17); preformed (C18) [44].

**Table 10:** Weight, cheese yield, and chemical composition (% DM) of cheese produced by control and treated ^1^ milk.

Item	CTR ^1^	WDGS + FFP ^1^	SEM	*p*-Value
Cheese, n	10	10		
Weight 24 h, kg	2.09	2.16	0.04	0.10
Cheese yield 24 h, %	9.48	9.82	0.19	0.10
Weight 3 mo, kg	1.748	1.763	0.03	0.65
Cheese yield 3 mo, %	7.95	8.01	0.20	0.69
Dry matter, %	69.98	69.56	0.43	0.50
Moisture, %	30.02	30.44	0.43	0.50
Fat, %	32.85	31.88	0.32	0.06
Protein, %	28.61	28.55	0.32	0.91

^1^ CTR: Control; WDGS + FFP: Condensed wheat distiller soluble + former foodstuff.

**Table 11:** Fatty acid profile in cheese fat produced with the two dietary treatments ^1^.

Fatty Acids (g/100 g FA)	CTR ^1^	WDGS + FFP ^1^	SEM	*p*-Value
C4:0	3.39	3.28	0.060	0.22
C6:0	1.98	1.89	0.042	0.15
C8:0	1.14	1.09	0.027	0.23
C10:0	2.52	2.42	0.060	0.25
C10:1 c9	0.23	0.23	0.008	0.49
C11:0	0.07	0.07	0.005	0.32
C12:0	2.90	2.81	0.066	0.32
C12:1 c11	0.03	0.03	0.001	0.51
C13:0	0.08	0.09	0.004	0.58
C14:0 iso	0.06 ^a^	0.05 ^b^	0.003	0.002
C14:0	9.00	8.71	0.107	0.07
C15:0 iso	0.15 ^a^	0.13 ^b^	0.005	0.003
C15:0 ante	0.41	0.39	0.012	0.33
C14:1 c9	0.83	0.85	0.019	0.55
C15:0	1.05	1.04	0.021	0.89
C16:0 iso	0.19	0.17	0.004	0.001
C16:0	37.90	35.80	0.750	0.07
C16:1t8	0.04	0.03	0.003	0.29
C16:1t9	0.04	0.04	0.002	0.73
C17:0 iso	0.30	0.30	0.007	1.00
C16:1c7	0.15	0.16	0.025	0.61
C16:1c9	1.88	1.89	0.039	0.83
C17:0 ante	0.52	0.52	0.012	0.85
C17:0	0.49	0.49	0.009	0.74
C17:1 c9	0.22	0.22	0.005	0.81
C17:1 c10	0.03	0.03	0.002	0.95
C18:0	6.94	7.32	0.197	0.19
C18:1 t6-8	0.25 ^a^	0.36 ^b^	0.016	<0.001
C18:1 t9	0.20 ^a^	0.27 ^b^	0.009	<0.001
C18:1 t10	0.57 ^a^	0.80 ^b^	0.049	0.005
C18:1 t11	0.66	0.72	0.030	0.13
C18:1 c6	0.46 ^a^	0.55 ^b^	0.010	<0.001
C18:1 c9	19.50 ^a^	21.10 ^b^	0.313	0.03
C18:1 t15	0.18 ^a^	0.22 ^b^	0.007	0.001
C18:1 c11	0.61	0.66	0.024	0.14
C18:1 c12	0.28 ^a^	0.36 ^b^	0.018	0.008
C18:1 c13	0.08 ^a^	0.10 ^b^	0.004	0.003
C18:1 t16	0.24	0.27	0.009	0.08
C18:2 t9t12	0.49	0.82	0.327	0.50
C18:2 cc	2.38	2.06	0.390	0.58
C20:0	0.11	0.16	0.058	0.54
C18:3n3	0.45	0.43	0.010	0.40
CLA9:11ct	0.38	0.37	0.061	0.92
C20:2n6	0.05	0.05	0.003	0.50
C22:0	0.04	0.05	0.007	0.39
C20:3n3	0.14	0.14	0.003	0.81
C20:4n6	0.17	0.16	0.004	0.81
C22:2	0.02	0.02	0.001	0.52
C24:0	0.03	0.03	0.002	0.10
**Groups of Fatty acids (g/100 g FA)**	**CTR ^1^**	**WDGS + FFP ^1^**	**SEM**	***p*-value**
OBCFA ^2^	3.32	3.24	0.054	0.30
SFA ^3^	69.27 ^a^	66.79 ^b^	0.457	0.002
MUFA ^4^	26.47 ^a^	28.90 ^b^	0.397	0.001
PUFA ^5^	4.07	4.06	0.136	0.96
n-3	0.58	0.57	0.011	0.47
n-6	2.92	2.93	0.812	0.92
De novo ^6^	23.84	23.06	0.357	0.14
Mixed ^6^	41.75	39.65	0.762	0.07
Preformed ^6^	34.22 ^a^	37.04 ^b^	0.582	0.004

^a,b^ Values within a row with different superscripts differ (*p* ≤ 0.05). ^1^ CTR: Control; WDGS + FFP: Condensed wheat distiller soluble + former foodstuff. ^2^ OBCFA: The sum of odd- and branched-chain fatty acids. ^3^ SFA: Short-chain fatty acids. ^4^ MUFA: Monounsaturated fatty acids. ^5^ PUFA: Polyunsaturated fatty acid. ^6^ De novo (from C4 to C14); mixed (C16. C16:1. C17); preformed (C18) [44].

**Table 12:** Environmental impact indicators calculated through LCA.

Environmental Indicators	CTR ^1^	WDGS + FFP ^1^
**Impact per kg of diet**		
^2^ GWP, kg CO_2_ eq.	0.74	0.55
Net fresh water, kg	22.0	15.20
Land occupation, m^2^a	0.99	0.75
**Impact per kg of milk produced**		
^2^ GWP, kg CO_2_ eq.	0.71	0.56
Net fresh water, kg	21.0	15.50
Land occupation, m^2^a	0.94	0.76

^1^ CTR: Control; WDGS + FFP: Condensed wheat distiller soluble + former foodstuff. ^2^ GWP: Global warming potential.

## Data Availability

The raw data supporting the conclusions of this article will be made available by the authors on request.

## Supplementary Materials

The following supporting information can be downloaded at https://www.mdpi.com/article/10.3390/ani15081113/s1. Table S1. Technological parameters of the cheesemaking process of the milk produced with two different treatments.
