# Supplementary material for: Former Food and Agro-Industrial By-Products in Dairy Cow Diets: Effects on Milk Quality and Cheese Production
Source: Animals (Basel). 2025 Apr 11;15(8):1113. doi: 10.3390/ani15081113 (PMC12024178; doi:10.3390/ani15081113)
Supplement: Supplementary file 1 [file animals-15-01113-s001.zip › animals-3515636-supplementary.pdf]

Table S1. Technological parameters of the cheesemaking process of the milk produced with two different treatments[1]

Item	CTR[1]	WDGS+FFP[1]
Milk temperature on farm,°C	5.13 ± 2.99	5.73 ± 2.63
Milk temperature in processing vat, °C	7.54 ± 2.11	6.09 ± 1.07
pH of Milk	6.76 ± 0.07	6.78 ± 0.08
°SH. ml/50ml	3.45 ± 0.10	3.46 ± 0.17
Temperature after addition of starters, °C	35.22 ± 0.33	35.13 ± 0.36
Temperature addition rennet, °C	35.46 ± 0.36	35.50 ± 0.29
pH of rennet	6.64 ± 0.03	6.64 ± 0.03
Resting period time, Min	18.00 ± 1.49	18.55 ± 1.47
Coagulation time, Min	25.30 ± 2.11	26.40 ± 2.70
Cutting process time, Min	3.05 ± 1.64	3.55 ± 2.21
Pre-heating at temperature of 43°C-44°C time, Min	2.15 ± 0.88	2.10 ± 0.97
Cooking Process time, Min	8.30 ± 2.25	7.70 ± 1.69
Temperature of cooking Process, °C	53.18 ± 0.63	53.19 ± 0.37
Duration of processing, Min	90.80 ± 5.79	91.10 ± 6.09
pH of whey	6.55 ± 0.05	6.55 ± 0.04

[1]CTR: control; WDGS+FFP: condensed wheat distiller soluble + former foodstuff.
